# Laboratory-Developed NGS Panel for Characterization of Advanced Colorectal Cancer, Real-World Data from a Tertiary Referral Centre

**DOI:** 10.3390/diagnostics16193091

**Published:** 2026-09-24

**Authors:** Floriana Jessica Di Paola, Giulia Calafato, Thais Maloberti, Laura Poppi, Giulia Ciccimarra, Sara Coluccelli, Viviana Sanza, Elisa Gruppioni, Annalisa Altimari, Antonio De Leo, Dario de Biase, Giovanni Tallini

**Affiliations:** 1 Biobank of Research, IRCCS Azienda Ospedaliero-Universitaria di Bologna, 40138 Bologna, Italy; florianajessica.dipaola@aosp.bo.it (F.J.D.P.); giulia.calafato@aosp.bo.it (G.C.); 2 Solid Tumor Molecular Pathology Laboratory, IRCCS Azienda Ospedaliero-Universitaria di Bologna, 40138 Bologna, Italy; thais.maloberti@aosp.bo.it (T.M.); laura.poppi@aosp.bo.it (L.P.); giulia.ciccimarra2@unibo.it (G.C.); sara.coluccelli@aosp.bo.it (S.C.); v.sanza@ausl.bologna.it (V.S.); elisa.gruppioni@aosp.bo.it (E.G.); annalisa.altimari@aosp.bo.it (A.A.); dario.debiase@unibo.it (D.d.B.); giovanni.tallini@unibo.it (G.T.); 3 Department of Medical and Surgical Sciences (DIMEC), University of Bologna, 40138 Bologna, Italy; 4 Department of Pharmacy and Biotechnology (FaBiT), University of Bologna, 40138 Bologna, Italy

**Keywords:** colorectal cancer, next-generation sequencing, *RAS* and *BRAF* mutations, molecular diagnostics, precision oncology

## Abstract

**Background/Objectives:** Colorectal cancer (CRC) is one of the most frequently diagnosed malignancies and a leading cause of cancer-related mortality worldwide. Its development and progression are driven by a complex interplay of genetic and epigenetic alterations that contribute to marked biological heterogeneity and variable clinical outcomes. Comprehensive molecular profiling is therefore essential for accurately characterizing CRC and guiding personalized therapeutic strategies. In this context, next-generation sequencing (NGS) has emerged as a powerful tool, enabling high-throughput, simultaneous detection of multiple clinically relevant genomic alterations in a single assay. **Methods:** We report our experience with a laboratory-designed NGS panel for the routine assessment of advanced-stage CRC. A total of 611 colorectal cancer specimens were analyzed using a laboratory-developed multi-gene NGS panel targeting 229 amplicons across 25 oncogenes and tumor suppressor genes. **Results:** The panel demonstrated analytical feasibility, with unsuccessful results observed in only 1.3% of the analyzed samples, due to low-quality/quantity DNA. *RAS* genes (*KRAS* and *NRAS*, altered in 51.2% and 2.7% of cases, respectively), together with *BRAF* (10.6%), *PIK3CA* (16.1%), *SMAD4* (11.8%), and *TP53* (64.3%), represented the most frequently altered molecular markers in the studied cohort. The frequency of major actionable genetic alterations in CRC was comparable between primary (*n* = 482) and metastatic (*n* = 121) lesions, suggesting similar mutation frequencies across the groups. **Conclusions:** This study presents a laboratory-developed multi-gene NGS panel specifically designed for molecular profiling of CRC in routine clinical practice.

## 1. Introduction

Worldwide, colorectal cancer (CRC) ranks as the third most commonly diagnosed malignancy and the second leading cause of cancer-related death [1]. Owing to global population growth, population aging, and socioeconomic development, the overall burden of CRC is expected to rise substantially, with projections estimating an additional 2.2 million new cases and 1.1 million cancer-related deaths by 2030 [2], and up to 3.2 million new diagnoses by 2040 [3]. CRC incidence remains highest in high-income regions, with a steady increase observed in middle- and low-income countries. Moreover, a rising trend in early-onset CRC has also been reported [3].

CRC is a biologically heterogeneous disease driven by recurrent somatic alterations that shape tumor behavior, influence therapeutic responses, and impact prognosis. Large-scale genomic studies, including The Cancer Genome Atlas (TCGA) and other sequencing efforts, have established that *KRAS* is the most frequently mutated oncogene in CRC, with mutations observed in approximately 35–50% of cases; the majority cluster in codons 12 and 13 of exon 2 and are well known to confer resistance to anti-EGFR therapies and are associated with poorer outcomes in metastatic settings [4,5,6]. *NRAS* mutations are less common, occurring in approximately 4–9% of CRCs, and, like KRAS alterations, predict a lack of benefit from EGFR inhibition [5,7]. *BRAF* mutations, most frequently p.Val600Glu, occur in approximately 4–15% of patients and define a distinct molecular subset characterized by an aggressive clinical course, worse overall survival, and frequent association with microsatellite instability (MSI) [8]. *PIK3CA* alterations, present in 10–25% of tumors, typically affect exons 9 and 20 and have been linked to modulation of PI3K/AKT signaling, potential impact on treatment response, and interaction with RAS pathway mutations [9,10,11,12]. *SMAD4* mutations, involved in TGF-β signaling, occur in a smaller subset (~5–15%) and are often associated with more advanced disease and metastatic progression, although their precise prognostic value remains an active area of research [13]. Additional structural and signaling genes, such as *TP53* and *APC*, are mutated at even higher frequencies and define critical events in colorectal tumorigenesis, though they are outside the core actionable set in most current therapeutic algorithms [14].

Routine molecular diagnostics in colorectal cancer (CRC) currently require testing for mismatch repair (MMR) protein expression and microsatellite instability (MSI) status, as well as *KRAS*, *NRAS*, and *BRAF* single-nucleotide point mutations. Assessment of MMR/MSI is critical to identify patients with defective DNA repair, as it not only has prognostic significance but also predicts response to immunotherapy. *KRAS*, *NRAS*, and *BRAF* mutation analysis informs the selection of targeted therapies, particularly anti-EGFR agents, and helps guide personalized systemic treatment strategies. However, relying on traditional single-gene testing is becoming increasingly challenging as more molecules are identified as potential therapeutic targets or mediators of treatment resistance. This growing complexity highlights the need for comprehensive molecular profiling approaches that can simultaneously assess multiple clinically relevant genes to guide personalized therapy and improve patient outcomes.

The advent of next-generation sequencing (NGS) has revolutionized this landscape by enabling simultaneous, high-throughput profiling of multiple genomic alterations in a single assay with high sensitivity and specificity [15,16]. Unlike single-gene PCR-based assays, NGS facilitates the detection of a broad spectrum of genomic events—including single-nucleotide variants (SNVs), insertions/deletions (indels), copy number changes, and fusions—while minimizing tissue consumption.

In this context, laboratory-developed NGS panels that integrate established biomarkers and emerging targets represent a resource-efficient and clinically informative strategy that enhances prognostic accuracy, elucidates mechanisms of therapeutic resistance, and expands patient eligibility for genotype-driven clinical approaches.

The present study evaluates the real-world analytical application of a laboratory-developed, in-house amplicon-based hotspot DNA NGS panel, designed for the detection of SNVs and small indels, in a large cohort of colorectal carcinoma specimens. The study focuses on the technical performance, feasibility, and robustness of the panel within a routine diagnostic workflow, as well as on the characterization of the mutational landscape identified across the analyzed cohort.

## 2. Materials and Methods

A total of 611 CRC cases were examined as part of routine diagnostic activity at the Molecular Pathology Laboratory of IRCCS Policlinico di S. Orsola, Bologna, Italy, between January 2023 and November 2025. All specimens were obtained from formalin-fixed, paraffin-embedded (FFPE) tissue blocks. DNA was extracted from two to three 10 μm thick sections, selected by a pathologist based on the most recent hematoxylin and eosin (H&E)-stained slide. A minimum tumor cell percentage of 10% was required for DNA extraction and subsequent molecular analysis. Tumor cell enrichment was estimated by a pathologist on the H&E-stained slide by assessing the proportion of neoplastic cells relative to the total number of cells in the marked area. DNA concentration was measured using a Qubit fluorometer (Thermo Fisher Scientific, Waltham, MA, USA). All the experimental procedures were carried out in accordance with the general authorization to process personal data for scientific research purposes from “The Italian Data Protection Authority” (http://www.garanteprivacy.it/web/guest/home/docweb/-/docwebdisplay/export/2485392 (accessed on 28 August 2026)). All information regarding human material was managed using anonymous numerical codes, and all samples were handled in compliance with the Helsinki Declaration (https://www.wma.net/fr/news-post/en-matierede-transfert-des-taches-la-securite-des-patients-et-la-qualitedes-soins-devraient-etre-primordiales/ accessed on 28 August 2026). The study did not affect the clinical management of the involved patients’ samples.

### NGS Analysis

Next-generation sequencing (NGS) analysis was performed using a custom multi-gene panel developed at the Molecular Pathology Laboratory of IRCCS Policlinico di S. Orsola [17]. This panel enables evaluation of SNVs and indels across 25 genes, encompassing 229 amplicons (15.04 kb, human reference genome hg19/GRCh37). The current panel configuration is based on the previously validated 22-gene assay, with modifications introduced to expand and refine the molecular targets assessed in clinical practice, as described by Maloberti et al. [18]. The analytical validation criteria and performance parameters established for the original assay were also applied to the current panel configuration. The genes and regions analyzed included: *BRAF* (exons 11 and 15), *CTNNB1* (exon 3), *EGFR* (exons 12, 18–21), *EIF1AX* (exons 1 and 2), *GNA11* (exons 4 and 5), *GNAQ* (exons 4 and 5), *GNAS* (exons 8 and 9), *H3F3A* (exon 1), *HRAS* (exons 2 and 3), *IDH1* and *IDH2* (exon 4), *KIT* (exons 8, 9, 11, 13, and 17), *KRAS* (exons 2–4), *MED12* (exons 1 and 2), *MET* (exons 2 and 14), *MYC* (exons 1–3), *NRAS* (exons 2–4), *PDGFRA* (exons 12, 14, and 18), *PIK3CA* (exons 10 and 21), *PTEN* (exon 5), *RET* (exons 5, 8, 10, 11, 13, 15, and 16), *RNF43* (exons 2 and 8), *SMAD4* (exons 6 and 9–12), *TERT* (promoter region, g.1295141–g.1295471), and *TP53* (exons 4–9). *APC* and *FBXW7* were not included in the panel because their mutations are widely distributed across the coding regions and are not restricted to a limited number of recurrent hotspot regions.

Sequencing was performed on the GeneStudio S5 Prime platform (Thermo Fisher Scientific, Waltham, MA, USA). Approximately 30 ng of DNA per sample was used to construct the amplicon library with the AmpliSeq Plus Library Kit 2.0. Template preparation was carried out using the Ion Chef system, followed by sequencing on an Ion 530 chip (Thermo Fisher Scientific, Waltham, MA, USA).

Sequence data were processed using Ion Reporter software (version 5.20, Thermo Fisher Scientific, Waltham, MA, USA). Variant filtering was applied with an allele frequency threshold ranging from 0.05 to 1.0. All filtered variants underwent manual review by two independent reviewers, and only those detected on both strands and present in at least 5% of total reads were included in the mutational analysis [17]. The assay routinely achieves a coverage uniformity of ≥95% and an on-target rate of ≥98%, with an analytical sensitivity/limit of detection of 5%. Variant pathogenicity was determined using the Varsome platform (https://varsome.com/, last accessed 30 November 2025). Variants classified as benign or likely benign were excluded from the analysis. For comparison with publicly available data, mutation frequencies from the TCGA PanCancer Atlas 2018 colorectal adenocarcinoma cohort were retrieved from cBioPortal (study ID: coadread_tcga_pan_can_atlas_2018), using the predefined cohort of 526 samples with mutation and CNA data.

Differences in mutation frequencies between primary and metastatic lesions were assessed using Fisher’s exact test with GraphPad Prism 9.0 (GraphPad Software, San Diego, CA, USA). *p*-values were adjusted for multiple comparisons using the Benjamini–Hochberg false discovery rate (FDR) procedure. Results were considered statistically significant at *p* < 0.05.

## 3. Results

Of the 611 samples analyzed by NGS, 8 (1.3%) were excluded due to low DNA quality or quantity. Consequently, subsequent analyses were conducted on the remaining 603 evaluable specimens. In only 44 out of 603 specimens (7.3%), no alterations were detected in any of the analyzed genes. In the remaining 559 specimens, a total of 1019 pathogenic, likely pathogenic, or variants of uncertain significance (P/LP/VUS) were detected across 23 genes (Table 1, Figure 1). No P/LP/VUS variants were observed in the other genes included in the NGS panel. *RAS* genes (*KRAS*, *NRAS*, and *HRAS*), along with *BRAF*, *PIK3CA*, *SMAD4*, and *TP53*, represented the most frequently altered molecular markers in the studied cohort (Table 1, Figure 1).

### 3.1. KRAS, NRAS, and HRAS

*KRAS* mutations were identified in 309 out of 603 specimens (51.2%) (Table 1, Figure 1). The majority of variants were located in exon 2 (273/309 mutated cases, 88.6%), while fewer alterations were detected in exon 3 (17/309, 5.5%) and exon 4 (19/309, 6.1%) (Figure 2A, Supplementary Table S1). The most common mutation was p.Gly12Asp (86/309 mutated cases, 27.8%), followed by p.Gly12Val (75/309, 24.2%), p.Gly13Asp (41/309, 13.3%), p.Gly12Cys (22/309, 7.1%), and p.Gly12Ala (21/309, 6.9%) (Figure 3A). All variants were classified as pathogenic or likely pathogenic, except for p.Asp108Asn, which was designated as a VUS according to the American College of Medical Genetics and Genomics (ACMG) criteria (Supplementary Table S1).

The *NRAS* gene was mutated in 16 of 603 specimens (2.7%) (Table 1, Figure 1). Most variants were in exon 3 (11/16, 68.8%), followed by those in exon 2 (5/16, 31.2%) (Figure 2B, Supplementary Table S2). The most common mutation was p.Gln61Lys (5/16, 31.3%), followed by p.Gln61Leu (3/16, 18.8%) and p.Gly13Arg (3/16, 18.8%). All variants were classified as pathogenic (Figure 3B, Supplementary Table S2).

*HRAS* mutations were found in 8 out of 603 specimens (1.3%) (Table 1, Figure 1). The identified variants were located in exons 2, 3, and 4 and were classified as pathogenic or VUS according to ACMG criteria (Supplementary Table S4).

### 3.2. BRAF

*BRAF* mutations were found in 64 out of 603 specimens (10.6%) (Table 1, Figure 1). The *BRAF* p.Val600Glu was the most frequent variant (50/64, 78.1%), followed by p.Asp594Gly (4/64, 6.3%), p.Asp594Asn (3/64, 4.7%), and other rarer *BRAF* variants (overall 7/64, 10.9%) (Figure 2C and Figure 3C, Supplementary Table S3). Nearly all variants were located in exon 15, except for p.Gly469Arg (*n* = 1), which was mapped to exon 11. All variants were classified as pathogenic (Supplementary Table S3).

### 3.3. Non-RAS/BRAF Mutations

Among the non-*RAS/BRAF* mutations, the most frequent alterations regarded the following genes: *PIK3CA* (97/603, 16.1%), *SMAD4* (71/603, 11.8%), and *TP53* (388/603, 64.3%) (Table 1, Figure 1 and Figure 2D,E). Most *PIK3CA* and *SMAD4* variants were classified as pathogenic or likely pathogenic, with only a few classified as VUS. The complete lists of variants for *PIK3CA* and *SMAD4* are provided in Supplementary Tables S5 and S6. *TP53* mutations spanned exons 4–9 and were nearly all classified as pathogenic. Additional, less frequent mutations were identified in several other genes, including *GNAS* (2.5%), *IDH1* (1.7%), *PTEN* (1.3%), *RET* (1.3%), *CTNNB1* (1.2%), *KIT* (0.8%), *MET* (0.8%), *MYC* (0.8%), *MED12* (0.7%), *GNAQ* (0.3%), *PDGFRA* (0.2%) (Table 1, Figure 1, Supplementary Tables S7–S17).

### 3.4. Primary and Metastatic Lesions

We next analyzed the distribution of major genetic alterations in primary (*n* = 482) and metastatic (*n* = 121) unmatched lesions (Table 2). Overall, the mutational profiles were largely comparable between the two groups, with a slightly higher, although not statistically significant, prevalence of alterations in primary lesions compared with metastatic specimens.

*KRAS* was the most frequently altered gene in both groups, with similar mutation rates in primary tumors (253/482, 52.5%) and metastatic samples (56/121, 46.3%) (FDR adjusted *p*-value = 0.393).

Mutations in *PIK3CA*, *SMAD4*, and *BRAF* were detected at comparable frequencies, although a modest reduction was observed in metastatic lesions. In contrast, *NRAS* and *HRAS* alterations were uncommon in both groups and were particularly rare in metastatic specimens (Table 2).

Overall, these findings indicate that the frequencies of the major driver alterations assessed were broadly similar between the primary and metastatic groups. However, given the unmatched nature of the samples and the limitations of targeted hotspot sequencing, these findings should not be interpreted as evidence of complete genomic equivalence between primary and metastatic tumors. Nevertheless, they support the use of primary tumor tissue for profiling the major driver alterations assessed by the panel when metastatic material is unavailable or inadequate.

## 4. Discussion and Conclusions

Currently, comprehensive characterization of the mutational landscape in colorectal cancer (CRC) is essential for guiding precision oncology approaches. Tumors harboring *RAS* wild-type status may derive clinical benefit from anti-EGFR monoclonal antibodies, whereas *RAS*-mutant CRCs are intrinsically resistant to these agents [19,20]. Patients with *BRAF* p.Val600Glu-mutated CRC represent a distinct molecular subgroup with poor prognosis but can benefit from combined BRAF and EGFR inhibition, as demonstrated in the BEACON CRC trial [21]. In addition, tumors characterized by microsatellite instability—high (MSI-H) or mismatch repair deficiency (dMMR) show marked sensitivity to immune checkpoint inhibitors, including pembrolizumab and nivolumab, which have become the standard of care in this setting [22,23]. A further actionable subgroup comprises HER2-amplified, RAS/BRAF wild-type CRCs, for which anti-HER2 strategies have demonstrated clinically meaningful activity [24]. Finally, although rare, *NTRK* gene fusions are targetable with tumor-agnostic TRK inhibitors [25]. Conversely, frequent alterations in *PIK3CA*, *TP53*, and *SMAD4* currently lack approved targeted therapies in CRC and are mainly of prognostic or biological relevance.

Currently, ESMO guidelines recommend testing for MMR status and for *KRAS*, *NRAS*, and *BRAF* mutations in all metastatic CRC patients at diagnosis, given their clinical relevance for treatment decisions [26,27,28]. In CRCs, all level I genomic alterations, as defined by the ESMO Scale for Clinical Actionability of Molecular Targets (ESCAT), are point mutations, and ESMO supports the use of NGS as an alternative to PCR-based methods.

Here, we report the routine clinical application of a laboratory-developed custom multigene NGS panel, which, compared with single-gene-based methods, offers several advantages, notably high-throughput multiplexing that enables simultaneous analysis of multiple genes and the detection of a broader spectrum of genomic alterations. Compared with our previous study [17], the present work includes a substantially larger cohort and provides a more comprehensive characterization of colorectal cancer molecular alterations, with a detailed description of variants identified across the full panel. This more comprehensive molecular characterization can yield valuable information for both clinical management and research, thereby improving prognostic stratification and deepening understanding of resistance mechanisms. Furthermore, broader implementation of NGS may enhance patient identification for clinical trials, thereby increasing access to innovative and potentially effective therapeutic options.

More importantly, in contrast to comprehensive genomic profiling approaches, such as broad commercial NGS panels, our assay was specifically designed as a focused hotspot panel to address the molecular alterations most relevant to routine CRC diagnostics, with a reduced target content that facilitates workflow scalability, data analysis, and cost containment while maintaining the analytical performance required for its intended clinical application.

The panel we developed for the molecular characterization of CRC is consistent with findings reported in the literature [4]. Indeed, *KRAS* was identified as the most frequently mutated driver gene, followed by *PIK3CA*, *SMAD4*, *BRAF*, and *NRAS*.

Uncommon mutations in genes such as *GNAS*, *IDH1*, *PTEN*, *CTNNB1*, *KIT*, *MET*, *RET*, and *GNAQ* have been reported in the studied CRC cohort using our custom NGS panel. Collectively, these rare mutations (mostly pathogenic or likely pathogenic according to ACMG criteria) were present in 13.2% of the analyzed cases, underscoring the value of broad multigene testing to identify potentially relevant genomic events beyond common driver mutations [29,30,31].

*GNAS* alterations have been associated with distinct clinicopathological features and, in selected studies, with survival outcomes [32,33], although their prognostic and predictive value remains to be fully validated. *PTEN* and *CTNNB1* alterations may reflect dysregulation of the PI3K/AKT and Wnt/β-catenin pathways, respectively, while rare *RET* alterations, particularly fusions, may identify potentially actionable tumors [31]. Other low-frequency alterations detected in our cohort, including *IDH1*, *KIT*, *MYC*, *MED12*, *GNAQ* and *PDGFRA*, have been reported in CRC genomic profiling studies, but their clinical significance remains poorly defined [31,34]. Overall, these findings highlight the molecular heterogeneity of CRC while underscoring that the prognostic or predictive value of most rare alterations remains insufficiently established.

Differences in mutation frequencies between the present cohort and TCGA should be interpreted considering methodological and cohort-related differences. Our study used targeted amplicon-based NGS, whereas TCGA primarily relied on whole-exome sequencing, resulting in differences in genomic coverage and variant detection. In addition, our cohort exclusively included advanced CRC cases, while TCGA comprised tumors from different disease stages. Geographic and ethnic differences may also contribute to variability in CRC mutational profiles. Finally, the use of FFPE-derived DNA in the present study may introduce pre-analytical factors related to DNA degradation and deamination, although targeted amplicon-based sequencing is generally well suited to the analysis of limited or fragmented DNA. Our routine workflow did not include specific measures to exclude FFPE-related artifacts; therefore, low-VAF variants, particularly C > T/G > A substitutions, and VUS should be interpreted with caution.

More importantly, this study demonstrates that the most frequent actionable genes are detectable in both primary and metastatic CRC lesions at nearly identical frequencies within the analyzed cohort. Although the comparison between primary tumors and metastatic lesions showed similar mutation frequencies for the major clinically relevant driver genes, our findings are based on independent (unmatched) cohorts and therefore reflect population-level concordance rather than patient-specific genomic concordance. Overall, our findings support the use of primary tumor tissue for routine molecular profiling of major clinically actionable driver genes when metastatic tissue is unavailable. However, primary and metastatic lesions should not be considered fully interchangeable, as intratumoral heterogeneity, clonal evolution, and treatment-emergent resistance mutations may result in additional alterations in metastatic sites. Therefore, matched primary-metastatic analyses remain important for assessing individual tumor evolution, clonal selection, and metastatic heterogeneity. Consistent with our findings, several comparative genomic studies have reported a high degree of concordance between the mutational profiles of primary and metastatic CRC lesions. In analyses of up to 95 paired primary-metastatic CRC samples, key driver genes—including *APC*, *KRAS*, *TP53*, *PIK3CA*, *SMAD4*, and *FBXW7*—displayed nearly identical mutation frequencies across tumor sites [35,36].

The custom NGS panel used in the present study was also demonstrated to be reliable and robust, failing to produce evaluable results in only 1.3% of the samples tested.

In addition, as demonstrated in our previously published studies, this panel enables the simultaneous analysis of multiple cancer types within a single sequencing run. It was specifically designed to address diagnostic, prognostic, and predictive clinical requirements not only for CRC, but also for other malignancies, including thyroid nodules, melanoma, pancreatic lesions, hepatocellular carcinomas, craniopharyngiomas, gliomas, and gastrointestinal stromal tumors (GISTs) [10,17,37].

Because the panel targets key genes shared across these tumor entities, it can be applied to the analysis of diverse tumor types in a single run, thereby reducing turnaround time and sequencing costs. Compared with commercially available multigene NGS panels, the optimized gene selection and cross-tumor applicability enable processing of a greater number of specimens per run. This level of flexibility is generally not achievable with tumor-specific commercial panels, while comprehensive commercial panels typically include extensive target regions, which limits sample throughput per run.

By pooling routine CRC specimens with samples from other tumor types, our workflow allows the analysis of up to 32–40 specimens per run, with a mean turnaround time of 7.4 working days. Reagent costs ranged from EUR 400 to 450 per sample, depending on run capacity. Compared with sequential single-gene testing, the multiplexed approach enables the simultaneous analysis of 25 genes in a single sequencing run, while requiring limited DNA input and reducing hands-on time and consumable use within our laboratory workflow. Overall, these features support the use of the NGS-based panel as a resource-efficient alternative to sequential single-gene testing. However, this represents a qualitative operational comparison based on our laboratory workflow and should not be interpreted as a formal health-economic or cost-effectiveness analysis.

We would like to clarify that the primary objective of the present study was to evaluate the application and routine clinical implementation of a laboratory-developed multigene NGS panel, rather than to assess its association with clinical outcomes. Consequently, analyses of treatment response, progression-free survival, overall survival, or treatment-specific predictive value were beyond the scope of this work. Nevertheless, the panel provides comprehensive molecular characterization of clinically relevant biomarkers to support routine molecular diagnostics and therapeutic decision-making according to current clinical guidelines. Further prospective studies with longitudinal clinical follow-up are warranted to establish the relationship between genomic alterations and patient outcomes.

This study has some limitations that should be acknowledged. First, the custom multigene NGS panel was specifically designed for hotspot mutation analysis of clinically relevant driver genes and does not detect other clinically relevant biomarkers in colorectal cancer, such as MMR/MSI status, HER2 amplification, or *NTRK* gene fusions, which require dedicated analytical methodologies. Therefore, comprehensive molecular characterization of CRC patients should integrate complementary diagnostic assays according to current clinical guidelines. Second, this study is a monocentric retrospective study; therefore, the absence of an independent external validation cohort may limit the generalizability of the findings. Future multicenter studies including independent validation cohorts will be warranted to further confirm the reproducibility and generalizability of our results. The absence of stratified subgroup analyses according to TNM stage, tumor differentiation grade, and MMR/MSI status represents a limitation of the present study, which was primarily designed to provide a descriptive characterization of the CRC mutational landscape and a methodological assessment of the NGS panel rather than a comprehensive clinicomolecular correlation analysis.

Overall, this study suggests that our laboratory-developed multigene NGS panel is a reliable tool for routine molecular profiling of colorectal cancer (CRC) and may support the implementation of precision medicine in clinical practice. Our data show that a targeted, resource-efficient multigene approach can provide clinically actionable information beyond standard single-gene testing without increasing turnaround time or costs (Figure 4). The simultaneous assessment of key predictive biomarkers enables a more comprehensive molecular stratification of patients, thereby supporting personalized therapeutic decision-making.

## Figures and Tables

**Figure 1:**
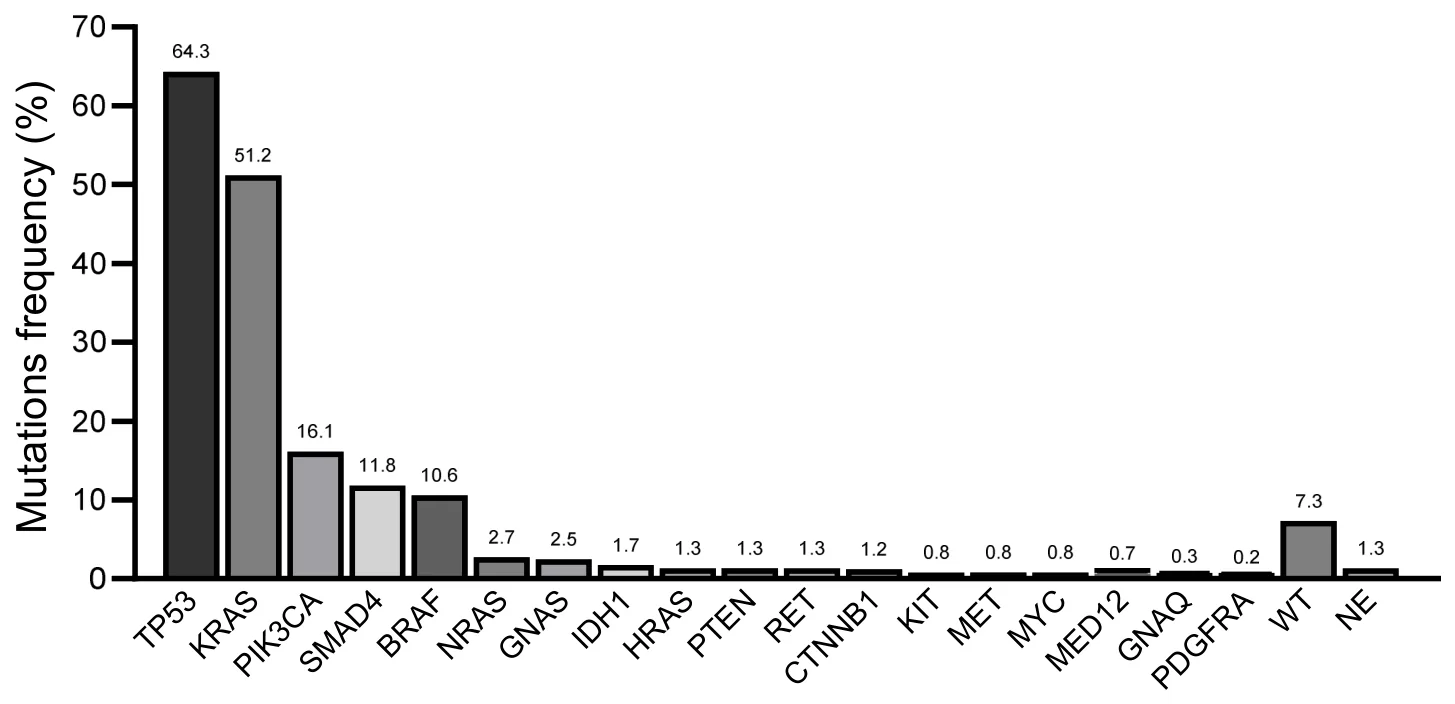
Frequency of mutations in the analyzed CRC cohort. WT: wild type; NE: not evaluable.

**Figure 2:**
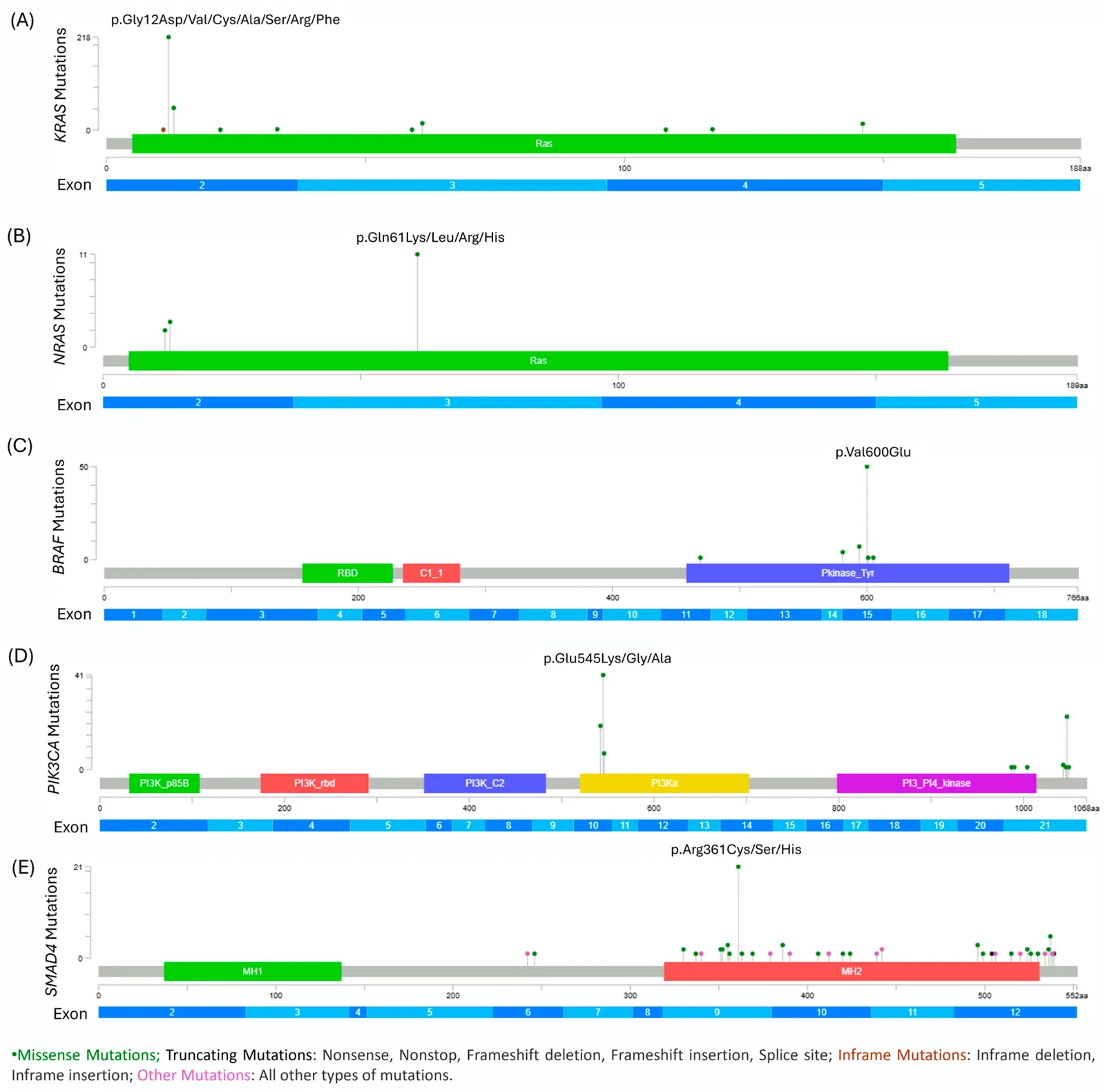
Distribution of genetic variants in *KRAS* (**A**), *NRAS* (**B**), *BRAF* (**C**), *PIK3CA* (**D**), and *SMAD4* (**E**) in the analyzed cohort.

**Figure 3:**
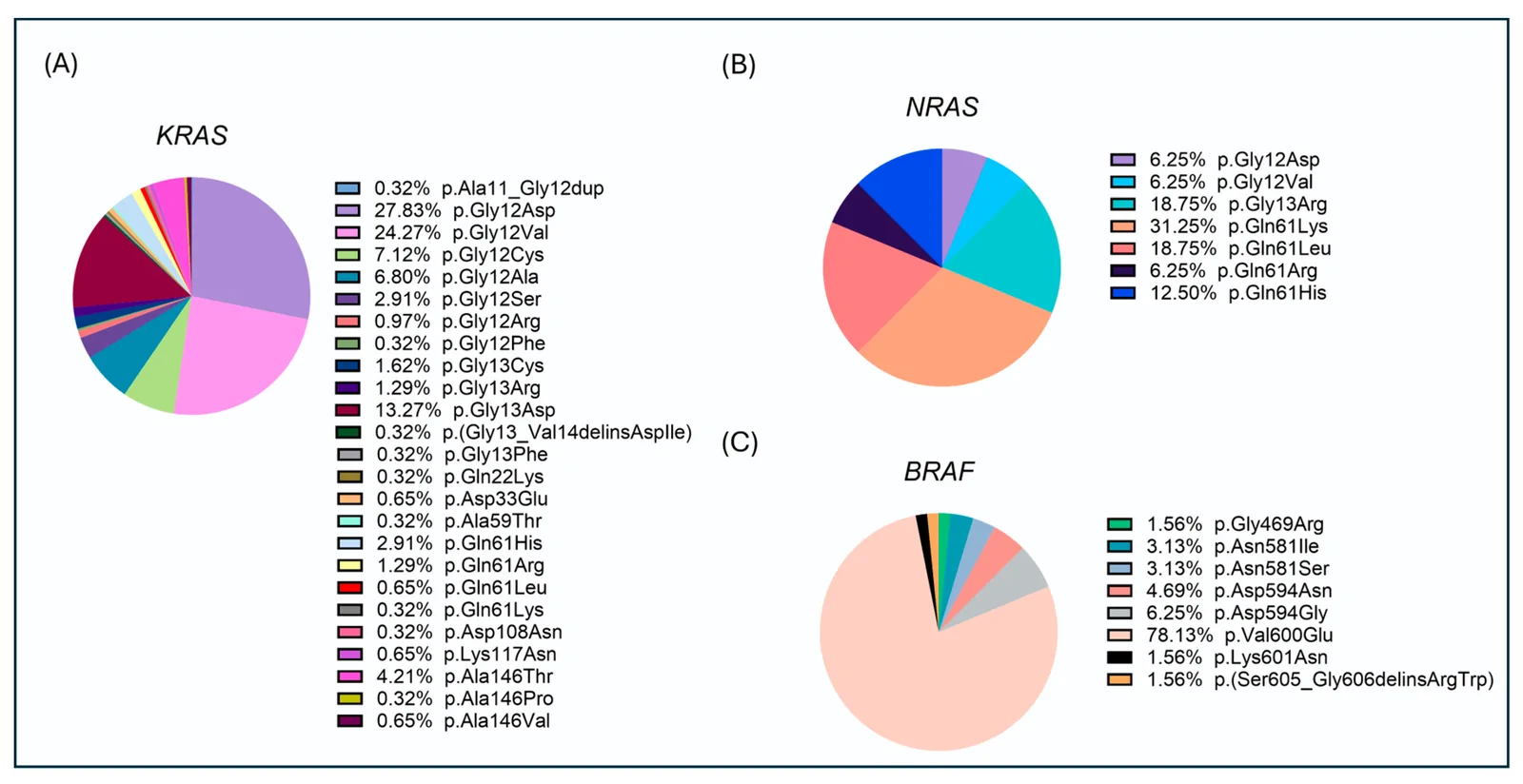
Distribution of protein-level variants in *KRAS* (**A**), *NRAS* (**B**), and *BRAF* (**C**) in the analyzed colorectal cancer cohort.

**Figure 4:**
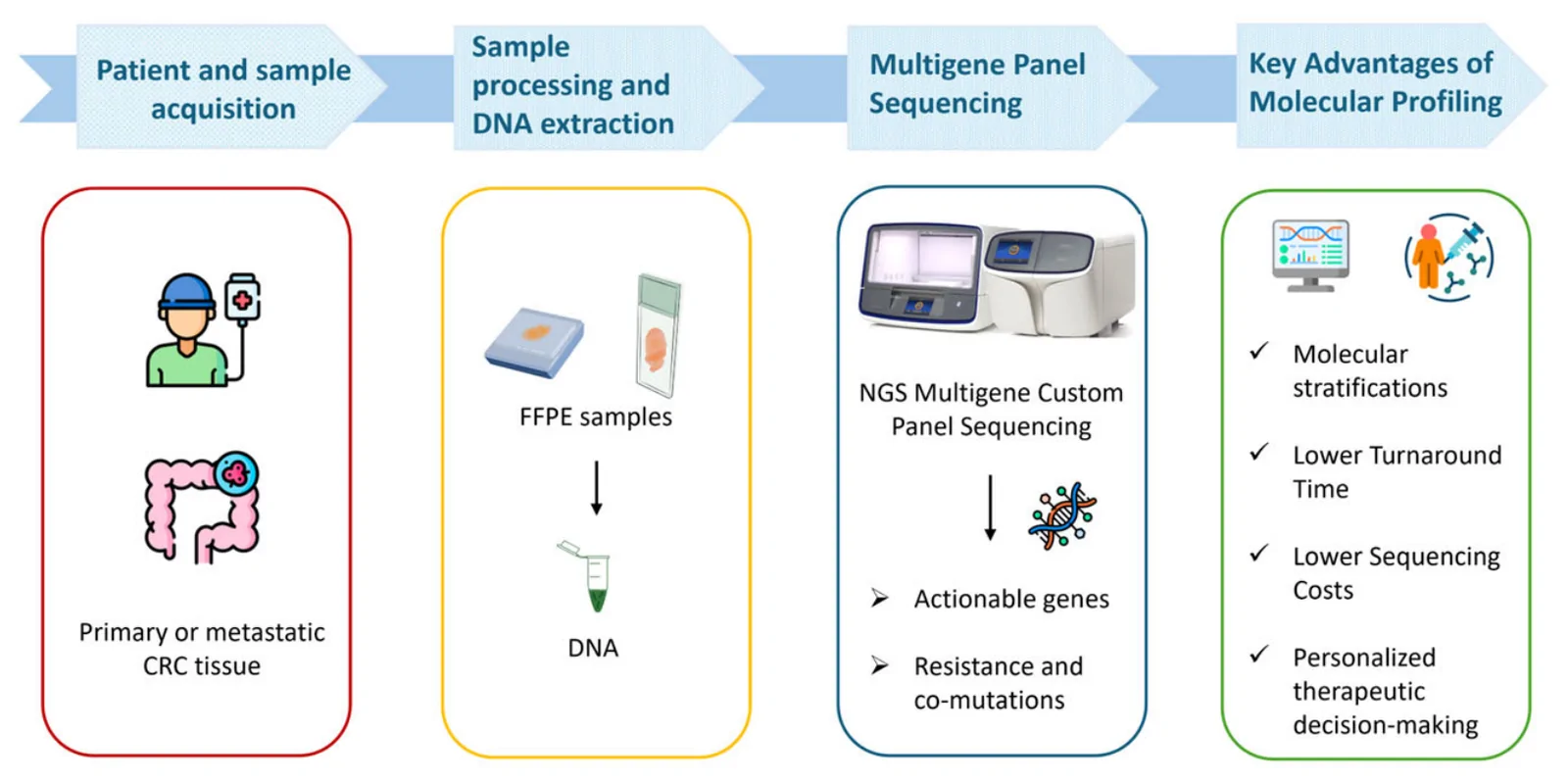
Schematic overview of the study design illustrating the development and the routine clinical application of a laboratory-developed multi-gene next-generation sequencing (NGS) panel. The workflow highlights its role in supporting molecular diagnostics and personalized management of colorectal cancer (CRC) patients in a routine clinical setting.

**Table 1:** Frequency of altered genes in the analyzed cohort and comparison with data obtained from TCGA.

Gene	Frequency in the Present Study (%) (Lab-Developed NGS Panel) (*n* = 603)	Frequency in TCGA (%) (Whole-Exome Sequencing) (*n* = 526)
*TP53*	64.3	60
*KRAS*	51.2	42
*PIK3CA*	16.1	28
*SMAD4*	11.8	16
*BRAF*	10.6	12
*NRAS*	2.7	7
*GNAS*	2.5	8
*IDH1*	1.7	2
*HRAS*	1.3	1
*PTEN*	1.3	9
*RET*	1.3	6
*CTNNB1*	1.2	7
*KIT*	0.8	5
*MET*	0.8	4
*MYC*	0.8	6
*MED12*	0.7	7
*GNAQ*	0.3	2
*PDGFRA*	0.2	6

**Table 2:** Frequency of driver gene mutations in primary and metastatic sites of the analyzed cohort.

Gene	Primary Lesion (%), *n* = 482	Metastasis (%), *n* = 121	FDR-Adjusted *p*-Value
*KRAS*	253 (52.5)	56 (46.3)	0.393
*NRAS*	15 (3.1)	1 (0.8)	0.393
*HRAS*	7 (1.5)	1 (0.8)	1.000
*BRAF*	53 (11.0)	11 (9.1)	0.727
*PIK3CA*	83 (17.2)	14 (11.6)	0.393
*SMAD4*	59 (12.2)	12 (9.9)	0.727
*TP53*	318 (66.0)	70 (57.9)	0.393

## Data Availability

The original contributions presented in this study are included in the article/Supplementary Material. Further inquiries can be directed to the corresponding author.

## Supplementary Materials

The following supporting information can be downloaded at: https://www.mdpi.com/article/10.3390/diagnostics16193091/s1, Table S1. *KRAS* variants in the analyzed cohort. Table S2. *NRAS* variants in the analyzed cohort. Table S3. *BRAF* variants in the analyzed cohort. Table S4. *HRAS* variants in the analyzed cohort. Table S5. *PIK3CA* variants in the analyzed cohort. Table S6. *SMAD4* variants in the analyzed cohort. Table S7. *GNAS* variants in the analyzed cohort. Table S8. *IDH1* variants in the analyzed cohort. Table S9. *PTEN* variants in the analyzed cohort. Table S10. *RET* variants in the analyzed cohort. Table S11. *CTNNB1* variants in the analyzed cohort. Table S12. *KIT* variants in the analyzed cohort. Table S13. *MET* variants in the analyzed cohort. Table S14. *MYC* variants in the analyzed cohort. Table S15. *MED12* variants in the analyzed cohort. Table S16. *GNAQ* variants in the analyzed cohort. Table S17. *PDGRFA* variant in the analyzed cohort.
