# Supplementary material for: Laboratory-Developed NGS Panel for Characterization of Advanced Colorectal Cancer, Real-World Data from a Tertiary Referral Centre
Source: Diagnostics (Basel). 2026 Sep 24;16(19):3091. doi: 10.3390/diagnostics16193091 (PMC13664408; doi:10.3390/diagnostics16193091)
Supplement: Supplementary file 1 [file diagnostics-16-03091-s001.zip › diagnostics-4354750-supplementary.pdf]

Supplementary Table S1. *KRAS* variants in the analyzed cohort.

KRAS (p.)	*KRAS (c.)*	*Exon*	*Cases (%), n = 309*	*ACMG Classification*
p.Ala11_Gly12dup	c.32_36dup	2	1 (0.3)	P
p.Gly12Asp	c.35G>A	2	86 (27.8)	P
p.Gly12Val	c.35G>T	2	75 (24.3)	P
p.Gly12Cys	c.34G>T	2	22 (7.1)	P
p.Gly12Ala	c.35G>C	2	21 (6.8)	P
p.Gly12Ser	c.34G>A	2	9 (2.9)	P
p.Gly12Arg	c.34G>C	2	3 (1.0)	P
p.Gly12Phe	c.34_35delinsTT	2	1 (0.3)	P
p.Gly13Cys	c.37G>T	2	5 (1.6)	P
p.Gly13Arg	c.37G>C	2	4 (1.3)	P
p.Gly13Asp	c.38G>A	2	41 (13.3)	P
p.(Gly13_Val14delinsAspIle)	c.[38G>A;40G>A]	2	1 (0.3)	P
p.Gly13Phe	c.38G>T	2	1 (0.3)	P
p.Gln22Lys	c.64C>A	2	1 (0.3)	LP
p.Asp33Glu	c.99T>G	2	2 (0.6)	LP
p.Ala59Thr	c.175G>A	3	1 (0.3)	P
p.Gln61His	c.183A>C	3	9 (2.9)	P
p.Gln61Arg	c.182A>G	3	4 (1.3)	P
p.Gln61Leu	c.182A>T	3	2 (0.6)	P
p.Gln61Lys	c.181C>A	3	1 (0.3)	P
p.Asp108Asn	c.322G>A	4	1 (0.3)	VUS
p.Lys117Asn	c.351A>C	4	2 (0.6)	P
p.Ala146Thr	c.436G>A	4	13 (4.2)	P
p.Ala146Pro	c.436G>C	4	1 (0.3)	P
p.Ala146Val	c.437C>T	4	2 (0.6)	P

ACMG: American College of Medical Genetics and Genomics; **P: Pathogenic, LP: Likely Pathogenic, VUS: Variant of Uncertain Significance.**

Supplementary Table S2. *NRAS* variants in the analyzed cohort.

NRAS (p.)	*NRAS (c.)*	*Exon*	*Cases (%), n = 16*	*ACMG Classification*
p.Gly12Asp	c.35G>A	2	1 (6.3)	P
p.Gly12Val	c.35G>T	2	1 (6.3)	P
p.Gly13Arg	c.37G>C	2	3 (18.8)	P
p.Gln61Lys	c.181C>A	3	5 (31.3)	P
p.Gln61Leu	c.182A>T	3	3 (18.8)	P
p.Gln61Arg	c.182A>G	3	1 (6.3)	P
p.Gln61His	c.183A>C	3	2 (12.5)	P

ACMG: American College of Medical Genetics and Genomics; **P: Pathogenic.**

Supplementary Table S3. *BRAF* variants in the analyzed cohort.

BRAF (p.)	*BRAF (c.)*	*Exon*	*Cases (%), n = 64*	*ACMG Classification*
p.Gly469Arg	c.1405G>C	11	1 (1.6)	P
p.Asn581Ile	c.1742A>T	15	2 (3.1)	P
p.Asn581Ser	c.1742A>G	15	2 (3.1)	P
p.Asp594Asn	c.1780G>A	15	3 (4.7)	P
p.Asp594Gly	c.1781A>G	15	4 (6.3)	P
p.Val600Glu	c.1799T>A	15	50 (78.1)	P
p.Lys601Asn	c.1803A>T	15	1 (1.6)	P
p.(Ser605_Gly606delinsArgTrp)	c.1813_1818delinsCGCTGG	15	1 (1.6)	P

ACMG: American College of Medical Genetics and Genomics; **P: Pathogenic**.

Supplementary Table S4. *HRAS* variants in the analyzed cohort.

HRAS (p.)	*HRAS (c.)*	*Exon*	*Cases (%), n = 8*	*ACMG Classification*
p.Gly13Arg	c.37G>C	2	1 (12.5)	P
p.Gly13Arg	c.37G>C	2	1 (12.5)	P
p.Arg41Gln	c.122G>A	3	1 (12.5)	VUS
p.Gly60Asp	c.179G>A	3	1 (12.5)	P
p.Gln61Arg	c.182A>G	3	1 (12.5)	P
p.Ala83Thr	c.247G>A	3	1 (12.5)	VUS
p.Ala134Thr	c.400G>A	4	2 (25)	VUS

P: Pathogenic, VUS: Variant of Uncertain Significance.

Supplementary Table S5. *PIK3CA* variants in the analyzed cohort.

PIK3CA (p.)	*PIK3CA (c.)*	*Exon*	*Cases (%), n = 97*	*Classificazione ACMG*
p.Glu542Lys	c.1624G>A	10	19 (19.6%)	P
p.Glu545Lys	c.1633G>A	10	31 (32%)	P
p.Glu545Gly	c.1634A>G	10	5 (5.2%)	P
p.Glu545Ala	c.1634A>C	10	4 (4.1%)	P
p.Glu545Asp	c.1635G>C	10	1 (1.0%)	P
p.Gln546Lys	c.1636C>A	10	5 (5.2%)	P
p.Gln546Glu	c.1636C>G	10	1 (1.0%)	P
p.Gln546Arg	c.1637A>G	10	1 (1.0%)	P
p.Ala987Ser	c.2959G>T	21	1 (1.0%)	VUS
p.Ala990Gly	c.2969C>G	21	1 (1.0%)	VUS
p.Met1004Ile	c.3012G>C	21	1 (1.0%)	LP
p.Met1043Ile	c.3129G>A	21	2 (2.1%)	P
p.Ala1046Thr	c.3136G>A	21	1 (1.0%)	LP
p.His1047Tyr	c.3139C>T	21	1 (1.0%)	P
p.His1047Arg	c.3140A>G	21	20 (20.6%)	P
p.His1047Leu	c.3140A>T	21	1 (1.0%)	P
p.His1047Gln	c.3141T>G	21	1 (1.0%)	P
p.Gly1049Arg	c.3145G>C	21	1 (1.0%)	LP

P: Pathogenic; LP: Likely Pathogenic; VUS: Variant of Uncertain Significance.

Supplementary Table S6. *SMAD4* variants in the analyzed cohort.

SMAD4 (p.)	*SMAD4 (c.)*	*Exon*	*Cases (%), n = 71*	*Classificazione ACMG*
p.Ser242Ter	c.725C>A	6	1 (1.4)	P
p.Pro246Thr	c.736C>A	6	1 (1.4)	VUS
p.Glu330Gln	c.988G>C	9	1 (1.4)	P
p.Glu330Gly	c.989A>G	9	1 (1.4)	P
p.Glu337Lys	c.1009G>A	9	1 (1.4)	LP
p.Lys340Ter	c.1018A>T	9	1 (1.4)	LP
p.Asp351Asn	c.1051G>A	9	1 (1.4)	P

SMAD4 (p.)	*SMAD4 (c.)*	*Exon*	*Cases (%), n = 71*	*Classificazione ACMG*
p.Asp351Val	c.1052A>T	9	1 (1.4)	P
p.Gly352Glu	c.1055G>A	9	1 (1.4)	P
p.Gly352Val	c.1055G>T	9	1 (1.4)	P
p.Asp355His	c.1063G>C	9	1 (1.4)	LP
p.Asp355Glu	c.1065C>A	9	2 (2.8)	LP
p.Pro356Ala	c.1066C>G	9	1 (1.4)	LP
p.Arg361Cys	c.1081C>T	9	7 (9.9)	P
p.Arg361Ser	c.1081C>A	9	2 (2.8)	P
p.Arg361His	c.1082G>A	9	12 (16.9)	P
p.Cys363Phe	c.1088G>T	9	1 (1.4)	LP
p.Asn369Asp	c.1105A>G	9	1 (1.4)	LP
p.Ala379GlufsTer6	c.1134_1135dup	9	1 (1.4)	LP
p.Gly386Arg	c.1156G>C	10	1 (1.4)	P
p.Gly386Ser	c.1156G>A	10	1 (1.4)	P
p.Gly386Val	c.1157G>T	10	1 (1.4)	P
p.Glu390Ter	c.1168G>T	10	1 (1.4)	P
p.Ala406Val	c.1217C>T	10	1 (1.4)	LP
p.Tyr412Ter	c.1236C>G	10	1 (1.4)	LP
p.Arg420Cys	c.1258C>T	10	1 (1.4)	P
p.Asp424Asn	c.1270G>A	10	1 (1.4)	VUS
p.Asp439AlafsTer53	c.1316_1320del	11	1 (1.4)	P
p.Gln442Ter	c.1324C>T	11	2 (2.8)	P
Splicing Site	c.1447+2T>C	–	1 (1.4)	P
p.Arg496His	c.1487G>A	12	2 (2.8)	LP
p.Arg496Pro	c.1487G>C	12	1 (1.4)	P
p.Cys499Tyr	c.1496G>A	12	1 (1.4)	LP
p.Ser504PhefsTer22	c.1510_1511del	12	1 (1.4)	P
p.Val506Ter	c.1516del	12	1 (1.4)	VUS
p.Arg515Ile	c.1544G>T	12	1 (1.4)	VUS
p.Glu520Ter	c.1558G>T	12	1 (1.4)	P
p.Trp524Arg	c.1570T>C	12	1 (1.4)	LP
p.Trp524Cys	c.1572G>T	12	1 (1.4)	P
p.Glu526Lys	c.1576G>A	12	1 (1.4)	LP
p.His530Gln	c.1590C>A	12	1 (1.4)	LP
p.Gln534Ter	c.1600C>T	12	1 (1.4)	P
p.Leu536Arg	c.1607T>G	12	1 (1.4)	LP
p.Leu536Pro	c.1607T>C	12	1 (1.4)	VUS
p.Asp537ArgfsTer39	c.1609_1610del	12	1 (1.4)	P
p.Asp537Tyr	c.1609G>T	12	1 (1.4)	VUS
p.Asp537Gly	c.1610A>G	12	2 (2.8)	LP
p.Asp537Glu	c.1611C>G	12	1 (1.4)	LP
p.Glu538Ter	c.1612G>T	12	1 (1.4)	P
p.Val539AspfsTer13	c.1616delT	12	1 (1.4)	LP

P: Pathogenic; LP: Likely Pathogenic; VUS: Variant of Uncertain Significance.

Supplementary Table S7. *GNAS* variants in the analyzed cohort.

GNAS (p.)	***GNAS (c.)***	***Exon***	***Cases (%), n = 15***	***ACMG classification***
p.Arg201Cys	c.601C>T	8	6 (40)	P
p.Arg201His	c.602G>A	8	9 (60)	P

P: Pathogenic.

Supplementary Table S8. *IDH1* variants in the analyzed cohort.

IDH1 (p.)	***IDH1 (c.)***	***Exon***	***Cases (%), n = 10***	***ACMG classification***
p.Thr106Met	c.317C>T	4	1 (10)	VUS
Splicing Site	c.123-2_123-1insT	—	1 (10)	VUS
p.Arg132Cys	c.394C>T	4	7 (70)	P
p.Ala134Thr	c.400G>A	4	1 (10)	VUS

P: Pathogenic; VUS: Variant of Uncertain Significance.

Supplementary Table S9. *PTEN* variants in the analyzed cohort.

PTEN (p.)	***PTEN (c.)***	***Exon***	***Cases (%), n = 8***	***ACMG classification***
p.Ala86Pro	c.256G>C	5	1 (12.5)	VUS
p.His93Tyr	c.277C>T	5	1 (12.5)	P
p.Val119Asp	c.356T>A	5	1 (12.5)	LP
p.His123Arg	c.368A>G	5	1 (12.5)	P
p.Arg130Ter	c.388C>T	5	2 (25.0)	P
p.Arg130Gln	c.389G>A	5	1 (12.5)	P
p.Arg142Gln	c.425G>A	5	1 (12.5)	LP

P: Pathogenic; LP: Likely Pathogenic; VUS: Variant of Uncertain Significance.

Supplementary Table S10. *RET* variants in the analyzed cohort.

RET (p.)	***RET (c.)***	***Exon***	***Cases (%), n = 8***	***ACMG classification***
p.Thr295Met	c.884C>T	5	1 (12.5)	VUS
p.Asp300Asn	c.898G>A	5	1 (12.5)	LP
p.Lys549Asn	c.1647A>C	8	1 (12.5)	LP
p.Val648Ile	c.1942G>A	11	1 (12.5)	VUS
p.Ala764Thr	c.2290G>A	13	1 (12.5)	VUS
p.Leu790Phe	c.2370G>T	13	1 (12.5)	P
p.Arg833His	c.2498G>A	14	1 (12.5)	VUS
p.Arg912Leu	c.2735G>T	15	1 (12.5)	VUS

P: Pathogenic; LP: Likely Pathogenic; VUS: Variant of Uncertain Significance.

Supplementary Table S11. *CTNNB1* variants in the analyzed cohort.

CTNNB1 (p.)	***CTNNB1 (c.)***	***Exon***	***Cases (%), n = 7***	***ACMG classification***
p.Ser33Cys	c.98C>G	3	1 (14.3)	LP
p.Ser33Pro	c.97T>C	3	1 (14.3)	LP
p.Gly34Glu	c.101G>A	3	1 (14.3)	LP
p.Ser37Pro	c.109T>C	3	1 (14.3)	LP
p.Ser45Phe	c.134C>T	3	1 (14.3)	P
p.Ser45Phe	c.134C>T	3	1 (14.3)	P
p.Ser45Pro	c.133T>C	3	1 (14.3)	LP

P: Pathogenic; LP: Likely Pathogenic; VUS: Variant of Uncertain Significance.

Supplementary Table S12. *KIT* variants in the analyzed cohort.

KIT (p.)	***KIT (c.)***	***Exon***	***Cases (%), n = 5***	***ACMG classification***
p.Met541Leu	c.1621A>C	10	1 (20.0)	VUS
p.Val560Asp	c.1679T>A	11	1 (20.0)	LP
p.Leu576Pro	c.1727T>C	11	1 (20.0)	P
p.Asp816His	c.2446G>C	17	1 (20.0)	LP
p.Asp816Val	c.2447A>T	17	1 (20.0)	LP

P: Pathogenic; LP: Likely Pathogenic; VUS: Variant of Uncertain Significance.

Supplementary Table S13. *MET* variants in the analyzed cohort.

MET (p.)	***MET (c.)***	***Exon***	***Cases (%), n = 5***	***ACMG classification***
p.Asp123Asn	c.367G>A	2	1 (20.0)	VUS
p.Thr230Met	c.689C>T	2	1 (20.0)	VUS
p.Gln318Ter	c.952C>T	2	1 (20.0)	LP
p.Ala319Thr	c.955G>A	2	1 (20.0)	VUS
p.Val1002LysfsTer15	c.3004_3005del	14	1 (20.0)	P

P: Pathogenic; LP: Likely Pathogenic; VUS: Variant of Uncertain Significance.

Supplementary Table S14. *MYC* variants in the analyzed cohort.

MYC (p.)	***MYC (c.)***	***Exon***	***Cases (%), n = 5***	***ACMG classification***
p.Ile140Phe	c.418A>T	2	1 (20.0)	VUS
p.Ser161Leu	c.482C>T	2	1 (20.0)	VUS
p.Glu270Lys	c.808G>A	3	1 (20.0)	VUS
p.Glu384Lys	c.1150G>A	3	1 (20.0)	VUS
p.Arg372Leu	c.1115G>T	3	1 (20.0)	VUS

VUS: Variant of Uncertain Significance.

Supplementary Table S15. *MED12* variants in the analyzed cohort.

MED12 (p.)	***MED12 (c.)***	***Exon***	***Cases (%), n = 4***	***ACMG classification***
p.Gly44Ser	c.130G>A	2	1 (25.0)	VUS
p.Gly44Cys	c.130G>T	2	1 (25.0)	VUS
p.Gly44Asp	c.131G>A	2	1 (25.0)	VUS
p.Gly44Val	c.131G>T	2	1 (25.0)	VUS

VUS: Variant of Uncertain Significance.

Supplementary Table S16. *GNAQ* variants in the analyzed cohort.

GNAQ (p.)	***GNAQ (c.)***	***Exon***	***Cases (%), n = 2***	***ACMG classification***
p.Gln209Pro	c.626A>C	5	1 (50.0)	LP
p.Gln209Leu	c.626A>T	5	1 (50.0)	P

P: Pathogenic; LP: Likely Pathogenic.

Supplementary Table S17. *PDGRFA* variant in the analyzed cohort.

PDGRFA (p.)	***PDGRFB (c.)***	***Exon***	***Cases (%), n = 1***	***ACMG classification***
p.Asp842_Met844del	c.2524_2532del	18	1 (100)	P

P: Pathogenic.
